# A Treatise on Fractures and Dislocations

**Published:** 1899-12

**Authors:** 


### Books and Magazines.

A Treatise on Fractures and Dislocations. For practitioners and students. By Lewis A. Stimson, B. A., M. D., Professor of Surgery in Cornell University Medical College, New York. In one octavo volume of 823 pages, with 321 engravings and twenty full page plates. Cloth, $5.00, net; leather, $6.00, net. Just ready. Lea Brothers & Co., Philadelphia and New York.

As a rule the very first surgical case the young doctor is called upon to treat is either a fracture or a dislocation, and a positive diagnosis as well as the best treatment to be adopted is to him a very difficult task. Indeed, the majority of physicians who have been in constant practice for many years find a case now and then which puzzles them severely in the matter of making a correct diagnosis; and every doctor who is called upon to do any surgery will gladly avail himself of every help in the treatment of this class of cases; hence the value of and necessity for this book.

This volume is, in a certain sense, a second edition of the author's two-volume work which was issued some years ago and is now out of print, but this volume is practically new, having been almost entirely rewritten to bring it up to date. The wider experience and the unusual hospital advantages which the author has had enables him to produce a work eminently suited to the wants of the student and the practicing physician. This work embraces the entire field of fractures and dislocations, the more important injuries, and those occurring most frequently in practice have received more attention and a wider discussion. A very valuable part of the volume is that given to the consideration of unreduced (ancient) dislocations and their management. Another of much interest and practical value is the chapter on delayed union, faulty union, failure of union. The volume is well supplied with illustrations, among them being a large number of X-ray full page plates. The book is a model of the bookmaker's art, and the publishers are to be congratulated on securing a work of so much practical interest to the profession, and on their excellent typographical work and the superior quality of materials entering into the make-up of the book. S. E. H.

